# A Moderated Mediation Model of Perceived Effect of Fitspiration Images on Self: The Influence of Media Literacy and BMI

**DOI:** 10.3390/ijerph19095077

**Published:** 2022-04-21

**Authors:** Ashraf Sadat Ahadzadeh, Shin Ling Wu, Fon Sim Ong, Ruolan Deng, Kam-Fong Lee

**Affiliations:** 1 Department of Journalism, Xiamen University, Sepang 43900, Malaysia; 2 Department of Psychology, School of Medical and Life Sciences, Sunway University, Petaling Jaya 47500, Malaysia; shinling_wu@hotmail.com; 3 Malaysian Research Institute on Ageing, Universiti Putra Malaysia, Serdang 43400, Malaysia; fonsim.ongfonsim@gmail.com; 4 Department of Communication, University of Vienna, 1010 Vienna, Austria; a11948895@unet.univie.ac.at; 5 Department of Education, UCSI University, Kuala Lumpur 56000, Malaysia; leekf@ucsiuniversity.edu.my

**Keywords:** internal locus of control, media literacy, perceived effect of media on self, attribution theory, fitspiration images, instagram

## Abstract

The present study investigated the relationship between internal locus of control and the perceived impact of Instagram fitspiration images on the self with media literacy as the mediating role in this relationship. This study also examined the importance of body mass index (BMI) as a moderating factor in the mediated model, where higher BMIs could weaken the relationship between internal locus of control and the perceived effect of fitspiration images mediated through media literacy. A sample of 321 Malaysian university students who were fitspiration viewers filled out a self-report questionnaire assessing internal locus of control, media literacy, perceived effect of fitspiration images on self, body satisfaction and BMI. The data analysis was performed using SPSS PROCESS macro. Results show that internal locus of control is negatively associated with the perceived impact of fitspiration images on self, mediated by media literacy. However, BMI moderates the mediated relationship such that the negative relationship between internal locus of control and the perceived effect of fitspiration images through media literacy does not exert an effect on those with high BMI. These results accentuate the value of incorporating a potential mediator and moderator into the direct relationship between internal locus of control and perceived effect of media ideals on self to provide an enhanced understanding of this process and offer practical insights about the protective role of media literacy and negative effects of high BMI.

## 1. Introduction

For years, the media has created a delusion that men and women should fit the narrowly defined standards of ideal societal appearance. In the increasingly digital world and the proliferation of social networking sites (SNS), Instagram has become one of the platforms for everyone to share appearance ideals using a hashtag, #fitspiration (a blended word combining ‘fitness’ and ‘inspiration’). Fitspiration became popular as the channel to promote healthy living focusing on diet and exercise [1,2], but it has eventually developed into a platform mainly hosting the images of thin bodies for females [1,3] and muscular or hypermuscular bodies for males [4], which are associated with an ideal image for many individuals [5,6,7,8,9,10].

A sociocultural perspective argues that perceived pressure from media (both traditional and digital media) to conform to the published ideals leads to the internalization of unrealistic body standards, upward comparison, unhealthy appearance schemas and appearance preoccupation [11,12,13,14,15]. Perceived pressure from media can be entangled with the perceived effects of media on self. While some individuals may perceive themselves susceptible to the media impacts, some others may believe that they are resistant to the content of media. According to attribution theory, the causes of individuals’ perceptions and behaviors can be derived from underlying properties including locus of control [16]. In view of this, individuals who believe in their own control over life are able to reduce the effect of media on self [17]. The assertion that locus of control contributes to the resistance to media effects has partly been substantiated in previous empirical research [18,19,20,21]. However, similar research with regards to the perceived effect of body ideals depicted on media, in specific fitspiration images on Instagram, has yet to be studied. It is presumed that internal locus of control may predispose individuals to resist the effects of fitspiration images. Therefore, examining the association between internal locus of control and perceived effects of fitspiration images on self is timely because much relevant research may afford an incomplete overview of the relationship.

In addition to the locus of control, which was found to be a defense to buffer the influence of media content on self, there could be other factors that ameliorate the deleterious impact of media messages. Therefore, this study further sheds light on the mechanism that may underlie the negative association between internal locus of control and the perceived effect of fitspiration images on self by introducing media literacy as a mediator. This postulate would also establish one of the main contributions of the present study in furtherance of the existing literature. Individuals may attribute their media literacy to their own abilities [16]. Besides, media literacy influences the acceptance or rejection of media content [22,23], and those with media literacy tend to reject undesirable media messages [24,25]. In light of this, the direct relationship of internal locus of control with the perceived effect of fitspiration images on self can be mediated by media literacy. Therefore, this study puts forth the assumption that engaging in critical scrutiny of media content via media literacy can meaningfully associate the influence of internal locus of control with the perceived effect of fitspiration images on self.

However, the mediation of media literacy might only exert an effect on the perceived impact of fitspiration images on self for viewers with low body mass index (BMI) [26,27]. Given this, the mediation model is further extended to a moderated mediation one, where BMI may possibly mitigate the negative relationship between internal locus of control and the perceived effect of fitspiration images mediated through media literacy. Therefore, we would presume that individuals with low BMI and high media literacy will not be affected by the fitspiration images regardless of the level of their internal locus of control. The present study also contributes further to the existing literature by examining whether high BMI curbs the mediation of media literacy in the association between internal locus of control and the perceived effect of fitspiration images on self.

Appearance-ideal internalization and appearance pressures are culture and context dependent [28]. Therefore, every context and culture is worth being explored, particularly if appearance pressure correlates with other variables, i.e., BMI and media literacy, that are the subjects of the present study. According to the World Health Organization (WHO) in 2019, “Malaysia has the highest rate of obesity and overweight among Asian countries with 64% of male and 65% of female population being either obese or overweight.” [29]. Evidence showed that obesity in Malaysia is associated with negative body image, fear of negative evaluation, low self-esteem and poor quality of life [30,31]. Furthermore, Malaysian young adults appeared to have low awareness of potential media effects and critical thinking abilities about the information they are exposed to in digital media, particularly on social media [32]. They were also found to struggle in evaluating the relevance and usefulness of information in digital media [33]. Such findings are worrying as about 86 percent of the Malaysian population (as of January 2021) was identified as active social media users [34], of which 13.8 million of them were Instagram users as of March 2021 [35]. Specifically, Malaysian Instagram users are dominantly young adults aged 18–34 [36]. Instagram gains its popularity among this group of Malaysians as it provides a platform for socialization, photo-sharing, entertainment, and visualized descriptions of products [37]. Evidence showed that a considerable percentage of Malaysian young adults are concerned with their body shape [38] and exposure to idealized images of the body on Instagram was found to exert an influence on their body image [11,39].

Given the above scenario, this study aims to test the relationship between internal locus of control and the perceived effect of Instagram fitspiration images on self, examine the mediating role of media literacy in this relationship, and explore the moderating effect of BMI on the mediated relationship between internal locus of control and perceived effect of Instagram fitspiration images on self in Malaysians young adults. The novelty of the present study lies in triangulating internal locus of control, media literacy and BMI to elucidate the underlying mechanism of perceived impact of fitspiration images on self, drawing upon attribution theory.

## 2. Theoretical Background and Hypotheses

### 2.1. Internal Locus of Control and Perceived Effect of Fitspiration Images on Self

An attribution is a causal explanation for an event of behavior [16]. Attribution theory posits that the perception of causality contributes to our subsequent behavior [16]. Those who believe that they are in control of the causes of the events perceive their performance is better in comparison to those who do not have such control, who will not perceive so. When individuals experience pleasant outcomes, attributions assist them to understand the causes of the events such that they can re-experience the events. On the other hand, when individuals experience undesirable outcomes, attributions help them to identify and circumvent the factors that resulted in the occurrence of the events [16]. Therefore, attributions help to shape emotional and behavioral responses.

Locus of control (LOC) is one of the major concepts in attribution theory where one believes that events in life are driven by his/her own actions (internal locus of control) or outside circumstances (external locus of control) [40]. Those who hold internal expectations are more likely to take the responsibility for their own actions as compared to those who hold external expectations [17]. Therefore, they believe that the outcome of their action is governed by their own abilities and drives. On the contrary, those who believe in chance and/or external powers are more likely to seek the attributes of their actions beyond their own abilities. According to attribution theory, people are motivated or desire to feel in control in order to maintain a positive self-image [16].

Attribution of internal locus of control can also navigate behaviors in different situations. Past research showed that individuals with higher levels of internal LOC are inclined to emphasize more on their body shape and evaluate themselves as confident in their ability to lose weight by exercising and dieting [41,42,43]. Internally oriented individuals also showed better body-areas satisfaction [44] and more positive perceptions of the health aspect of their body images [45]. Internal locus of control was also found to be associated with media effect on self. A survey study showed that the more people viewed news about terrorism shown in the media involuntarily and the lower their internal locus of control, the more vulnerable they were to develop post-traumatic stress disorder (PTSD) symptoms in missile attacks [18]. Likewise, those who believed in external forces beyond their own control such as chance, fate, or luck reported watching more television programs resulted in being more pessimistic about society than those who believed in internal forces controlling the course of their own lives [19]. Additionally, individuals who scored high on internal locus of control reported lower perceived internet and social media addiction [20,46]. Similarly, they also reported a high level of confidence in socializing online and resisting online peer pressure [47].

The power of the posts on Instagram is identified through hashtags [48]. Research showed that users are more likely to “use the same, with the owner, hashtags to annotate an image” on Instagram [49]. Besides, more and more social media platforms support hashtags to facilitate information classification. Comito et al. [50] identified hashtag-based clustering as one of the streaming clustering techniques for topic detection in social data streams. Likewise, research found that the use of hashtags helps to determine the sentiments, preferences and topics of the post [51,52]. As such, the hashtag fitspiration on Instagram has become a popular social media trend for sharing fitness-related content. The power of this hashtag can be best harnessed to improve users’ health, tailoring content to match user preferences. However, personality characteristics could also be the critical determinant of Instagram users’ exposure to fitspiration images. Possessing an internal locus of control could be associated with less attention paid to images on fitspiration, less disposition made to internalize the bodies portrayed in fitspiration images. Indeed, the viewers of fitspiration images who believe that they could determine their own actions and behaviors are less likely to evaluate the effects of fitspiration images. In contrast, those viewers who disbelieve that they could determine their own actions are more susceptible to the effects of images. Therefore, it can be postulated that those who believe that their physical appearance is under their own control might evaluate themselves as having the defense mechanism against the impact of fitspiration images on Instagram. Based on the premise of attribution theory and past empirical results, the following hypothesis is put forth:

**Hypothesis** **1.** *There is a significant negative relationship between internal locus of control and perceived impact of fitspiration images on self.*

### 2.2. Mediation of Media Literacy 

Although theoretical and empirical findings have supported the notion that internal locus of control is associated with the perceived impact of media, the underlying mechanism of this association and the possible influence of third variables has not been explored. Studies showed that individuals with a perception of personal control over their actions tend to have more critical views [53], including having control over media content effects. In other words, to them, having a personal locus, “the set of needs, drives and intellectual abilities,” helps them to process and to decode the information they have accessed [54]. Therefore, this study intends to introduce media literacy as a mediator and test the mediation effect of media literacy.

Media literacy is defined as “understanding the mechanics of messages, analyzing and evaluating their meaning, and assigning them value” [55]. According to the message interpretation process model, media literacy entails message interpretation and skepticism which aid in either accepting or rejecting a message [56]. Media literacy with an emphasis on critical thinking is able to challenge the influence of media content [57,58]. There is widespread evidence of the protective effect of media literacy on media exposure to ideal appearance on social media [22,24,25,59]. Thus, media literacy empowers people against unrealistic media images by engaging in active filtration and eventually rejecting images that they perceive to be unrealistic or undesirable [22,23]. In other words, media literacy can reduce body image concerns and body image disturbance by averting the effect of the social comparison process [24,25,60,61].

In light of the empirical results reviewed above, a mediated model can be proposed to examine the relationship between internal locus of control and the perceived impact of media on the self through media literacy. Fitspiration viewers with higher scores on internal locus of control are more likely to report higher scores on media literacy [53,54,62] which in turn contribute to the reduced perceived impact of media on self. In fact, media literacy rationalizes the indirect relationship between internal locus of control and perception of media effect on self. By engaging in critical viewing and questioning the fitspiration images, individuals may not engage in comparing their bodies with those displayed in fitspiration [24,60]. Therefore, it can be deduced that higher levels of media literacy reduce the perceived effect of fitspiration images on self. Based on the literature reviewed above, the following hypothesis is proposed:

**Hypothesis** **2.** *Media literacy mediates the negative relationship between internal locus of control and perceived impact of fitspiration images on self.*

### 2.3. Moderation of BMI

Although internal locus of control may affect the perceived influence of fitspiration images on self, there is a likelihood that not all viewers are affected by fitspiration images. There could be a third factor that influences the effect of fitspiration images notwithstanding the level of locus of control or media literacy. A possible moderator that may mitigate the association between internal locus of control and the perceived effect of fitspiration images on self is BMI.

The importance of physical state, specifically BMI, in the development of body image has been substantiated in the bourgeoning literature. A higher level of BMI leads to body dissatisfaction and unhealthy behaviors (e.g., eating disorders, bulimia, etc.) [15,63,64,65]. Research showed that a high level of BMI exacerbates the negative effects of media idealized images [66]. Individuals with high BMI are more likely to feel pressured to meet the standards of ideal bodies depicted in media [15,27,67,68], thus struggling with a sense of body dissatisfaction, social anxiety and social phobia [69,70]. Results also showed that BMI is positively correlated with the internalization of media body ideals [14,27].

Fitspiration viewers who have lower BMI are more likely to leverage their media literacy, which in turn reduces the perceived effect of fitspiration images. They are inclined to perceive themselves as not susceptible to fitspiration images because they tend to have no concern about their weight and do not engage in comparison with bodies in fitspiration images. In contrast, those with high BMI are more likely to be affected by fitspiration images even though they are media literate. In other words, a high level of media literacy will not necessarily furnish fitspiration viewers to translate their critical stance into resistance to fitspiration image effects due to the influence of BMI.

Based on this assumption and the reviewed literature, the present study examined whether the indirect association between internal locus of control and the perceived effect of images on self could be moderated by BMI. Therefore, the following hypothesis is developed:

**Hypothesis** **3.** *BMI moderates the negative relationship between media literacy and the perceived impact of fitspiration images on self, with the relationship being stronger for those with lower BMI.*

The mediation effect of media literacy is further hypothesized to be moderated by BMI. Media literacy fails to translate internal locus of control into perceived effect of fitspiration images on self when BMI is high. An extensive literature has established the link between locus of control and obesity [71,72]. External locus of control was found to be a provoking factor for developing overweight [73]. In brief, those with high BMI are more likely to believe that events in life are beyond their control. Furthermore, research demonstrated that higher BMI is associated with decreased perception of one’s ability to think critically, and those who exercised reported having stronger critical thinking skills compared to those who exercised less frequently [74]. High BMI also makes individuals susceptible to the adverse effects of idealized images depicted in media [15,66,68]. All these findings consistently prove that high BMI can be a risk factor influencing individuals’ resistance to idealized media images, their critical thinking and belief in their own power over the events in their life. Underlying the existing empirical evidence, this study further postulates that the mediating role of media literacy on the negative relationship between internal locus of control and perceived impact of fitspiration images is subject to the fitspiration viewers’ BMI. Indeed, the mediating effect of media literacy will only exert its influence on the negative effect of internal locus of control on the perceived impact of fitspiration image on self for individuals who have low BMI. To test this, the following hypothesis is proposed:

**Hypothesis** **4.** *BMI moderates the mediating effect of media literacy in the relationship between internal locus of control and the perceived impact of fitspiration images on self.*

## 3. Method

### 3.1. Participants

A total of 321 participants between the age of 18–31 years old (Mean = 20.93, SD = 2.18) were included in this research. Among them, 72.9% were female and 60.7% were Chinese. The participants’ BMI ranged from 13.15 to 38.58 (Mean = 21.40, SD = 3.85). The demographic profile of the participants is displayed in Table 1.

### 3.2. Research Procedure and Data Collection

A cross-sectional survey was conducted to collect data from Malaysian young adult Instagram users. Three private universities located in Klang Valley, Malaysia, were targeted as the research setting. Malaysian Instagram users are predominantly young adults aged 18–34 [36] who are mainly reachable in higher education institutions. A research assistant was recruited to collect data using the convenience sampling method. A “drop and collect” method was adopted to collect data. Babbie [75] commented that when a research worker either delivers the questionnaire, picks it up, or both, the completion rate seems to be higher than for a straightforward mail survey. The research assistant personally began to distribute the questionnaires among students accessible in the library and café spaces in the universities. After explaining the research topic and its objectives, students who agreed to participate in the research study were distributed a copy of the survey. The majority of the participants completed the survey instantly while a small group of them requested to return the filled-in survey within a couple of hours. The research assistant was on standby during the session to respond to the participants’ probable questions and to collect the filled-in survey. The first section of the questionnaire consisted of the informed consent form where participants were ensured of the confidentiality of their responses and that their participation in this study was completely voluntary. Therefore, they had the right to withdraw from participation at any time. The same section provided participants with information about the research objectives. The next section covered questions on participants’ demographic characteristics, followed by questions about their activities on Instagram (Meta Platforms, Menlo Park, CA, United States). This section ended with a filter question asking if participants view fitspiration images on Instagram (1 = Never, 2 = Rarely, 3 = Sometimes, 4 = Often, 5 = Always). The filter question was accompanied by a brief explanation about fitspiration images on Instagram. Visiting fitspiration images on Instagram was the screening criterion for exclusion from analysis. Since users could show a differential sensitivity depending on their frequency of exposure to fitspiration images, there was a potential concern that users who never or rarely viewed fitspiration images could alter the pattern of sensitivity shown in comparison to users who sometimes, often, and always view fitspiration images. Therefore, we decided to exclude participants who ‘rarely’ or ‘never’ viewed fitspiration photos from the analysis. The following sections of the questionnaire sought information about independent and dependent constructs. Questionnaires with more than 10% missing responses were also excluded from the analysis. Given these criteria, out of 440 questionnaires collected conveniently, only 321 responses were included in the analysis. Before conducting the study, we sought approval from the Ethics Committee of Xiamen University, Malaysia. The committee approved the protocol of the study including the research procedure, the rights and safety of the participants, and the method of data collection (Reference number: REC-1912.01). The data collection took two months from 10 January 2020 to 28 February 2020.

### 3.3. Measurements

#### 3.3.1. Perceived Effect of Fitspiration Images on Self

Following Wan et al.’s [76] research, the respondents were asked to estimate the impact of fitspiration images on Instagram on self, using four items. Items were “fitspiration images on Instagram have a powerful impact on me”; “Seeing fitspiration images on Instagram makes me feel that boys/girls should have the same idealized body”; “Seeing fitspiration images on Instagram influences my perception of how other boys/girls should look” and “Seeing fitspiration images on Instagram makes me feel less satisfied with how they look.” For each item, respondents indicated their level of agreement using a 5-point scale ranging from 1 = strongly disagree to 5 = strongly agree. Wan et al.’s [76] study demonstrated an acceptable level of reliability for the perceived impact of advertising models on the self (α = 0.88). For this study, the four items assessing the perceived impact of fitspiration images on self achieved an acceptable level of reliability (α = 0.82).

#### 3.3.2. Internal Locus of Control

We employed a brief version of Levenson’s locus of control scale constructed by Sapp and Harrod [77]. The scale comprised nine items; three items measuring internal control (e.g., my life is determined by my own actions), three items measuring powerful control by others (e.g., my life is chiefly controlled by powerful others), and three items measuring chance control (e.g., when I get what I want, it’s usually because I’m lucky) [77]. Responses were rated on a 5-point Likert-type scale (from 1 = strongly disagree to 5 = strongly agree). In order to estimate the participants’ internal locus of control, we followed previous research where responses to the powerful others and chance control items were recoded and added up to the score of internal control; so that higher scores reflected greater internal control [78]. The level of internal consistency for the internal locus of control obtained in this study was acceptable (α = 72).

#### 3.3.3. Media Literacy

A 10-item media attitudes questionnaire (MAQ) was used to assess participants’ media literacy [79]. A number of past studies used the same scale to measure media literacy [24,80,81], supporting the appropriateness and validity of the scale. For the purpose of this study, we adapted the items of the scale to provide a suitable context for the items. Changes to the item context is one of the common scale adaptation methods [82]. This method involves changes in the situational specificity of the items while maintaining the essence of the items in the scale. For example, the word “ads” was changed to “people in Instagram fitspiration photos”. Some items included “I could be as thin as people in Instagram fitspiration photos,” “people in Instagram fitspiration photos have perfect bodies,” “Normally women (in real life) are as thin as women in Instagram fitspiration photos.” Responses were rated on a 5-point Likert-type scale (from 1 = strongly disagree to 5 = strongly agree). The level of internal consistency obtained in this study for the scale was acceptable (α = 0.70).

#### 3.3.4. BMI

Participants’ self-reported weight (g) and height (cm) were used to calculate the BMI, a ratio of weight to height. This method was appropriate as previous research utilized self-reported weight and height to determine the BMI score [68].

#### 3.3.5. Body Satisfaction

The respondents were asked to rate their satisfaction with four parts of their body using a 5-point Likert scale where 1 refers to “strongly dissatisfied” and 5 refers to “strongly satisfied”. These four parts of the body were the stomach, hips, buttocks, and thighs. These four parts were included as they are more highlighted in fitspiration images [4]. These four items demonstrated an acceptable level of reliability (α = 0.84).

### 3.4. Data Analysis

The data were analyzed using IBM SPSS software version 26 (IBM Corp., Armonk, NY, USA). Pearson correlation was used to test Hypothesis 1 to determine the relationship between internal locus of control and the perceived impact of fitspiration images on self. Hypothesis 2 hypothesized that media literacy mediates the negative relationship between internal locus of control and the perceived impact of fitspiration images on self. The two hypotheses were tested using the mediation model (Model 4) of the SPSS PCOCESS macro [83]. Meanwhile, Hypothesis 3 about BMI moderating the negative relationship between media literacy and the perceived impact of fitspiration images on self, with the relationship being stronger for those with lower BMI, as well as Hypothesis 4 about BMI moderating the mediating effect of media literacy in the relationship between internal locus of control and perceived impact of fitspiration images on self, were tested using the moderated mediation model (Model 14) of the SPSS PCOCESS macro [83]. Age, gender and body satisfaction were controlled in all analyses. A bootstrapping of 5000 resamplings was performed with a confidence interval of 95%. The mediation and moderation analysis are considered significant when zero does not appear in the 95% confidence interval result. SPSS PROCESS was used because bootstrapped confidence intervals are now the standard for testing indirect effects. It has an increase in power compared to the Sobel test [83].

## 4. Results

The mean, standard deviation and correlation value for all variables are shown in Table 2. There was a significant negative relationship between internal locus of control and perceived impact of fitspiration images on self (*r* = −0.20, *p* < 0.001), supporting Hypothesis 1. Internal locus of control also correlated significantly and positively with media literacy (*r* = 0.21, *p* < 0.001). Media literacy was found to have a significant negative relationship with the perceived impact of fitspiration images on self (*r* = −0.21, *p* < 0.001). No significant relationships were found between age, BMI and body satisfaction with the perceived impact of fitspiration images on self.

Hypothesis 2 hypothesized that media literacy mediates the negative relationship between internal locus of control and perceived impact of fitspiration images on self. The effect of age, gender and body satisfaction were controlled. The total effect of internal locus of control on the perceived impact of fitspiration images on self was found significant, where B = −0.14, *t*(316) = −3.68, *p* < 0.001. Internal locus of control had a significant direct effect on media literacy, where B = 0.23, *t*(316) = 3.81, *p* < 0.001, while media literacy has a significant direct effect on perceived impact of fitspiration images on self, where B = −0.11, *t*(315) = −3.31, *p* = 0.001. The direct effect of internal locus of control on the perceived impact of fitspiration images on self while controlling for age, gender, body satisfaction and media literacy was significant, where B = −0.11, *t*(315) = −2.96, *p* = 0.003. The indirect effect of internal locus of control on the perceived impact of fitspiration images on self via media literacy was found significant, where indirect effect = −0.03, SE = 0.01, 95% CI (−0.06, −0.01). This finding supported Hypothesis 2 in which media literacy significantly mediated the negative relationship between internal locus of control and perceived impact of fitspiration images on self.

**Figure 1:**
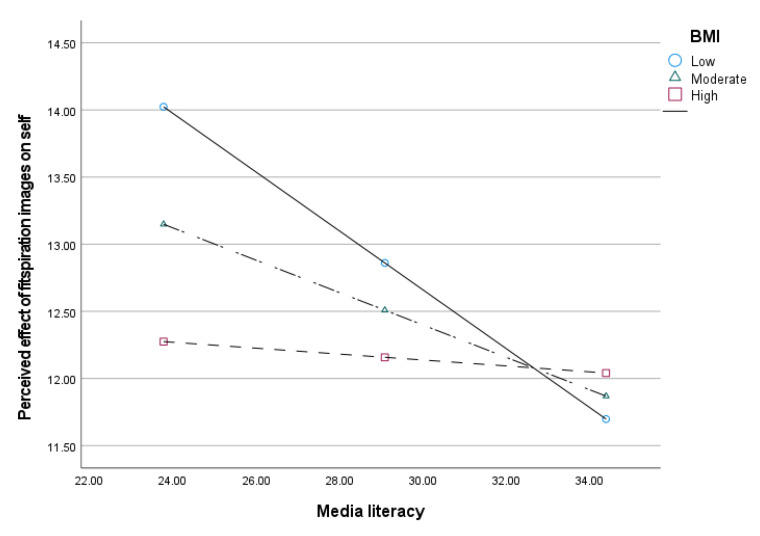
Interaction effect between media literacy and BMI on the perceived effect of fitspiration images on self.

Hypothesis 3 and 4 were analyzed using the moderated mediation model (Model 14) in PROCESS macro while controlling for age, gender and body satisfaction. Table 3 shows that internal locus of control had a significant direct effect on media literacy, where B = 0.23, *t*(316) = 3.81, *p* < 0.001, and perceived impact of fitspiration images on self, where B = −0.11, *t*(313) = −3.08, *p* = 0.002. The direct effect of media literacy on the perceived impact of fitspiration images on self was significant, where B = −0.67, *t*(313) = −3.65, *p* < 0.001. The association between media literacy and the perceived impact of fitspiration images on self was significantly moderated by BMI which supported Hypothesis 3, where B = 0.03, *t*(313) = 3.07, *p* = 0.002. Based on the conditional effects, the association between media literacy and the perceived impact of fitspiration images on self was found significant for low and moderate BMI, but not significant for participants with high BMI. Participants with low, where B = −0.022, SE = 0.05, 95% CI (0.31, −0.13), and moderate BMI, where B = −0.12, SE = 0.03, 95% CI (−0.19, −0.05), are more likely to have a higher perceived effect of fitspiration images on self when media literacy is low (see Table 4 and Figure 1).

**Figure 2:**
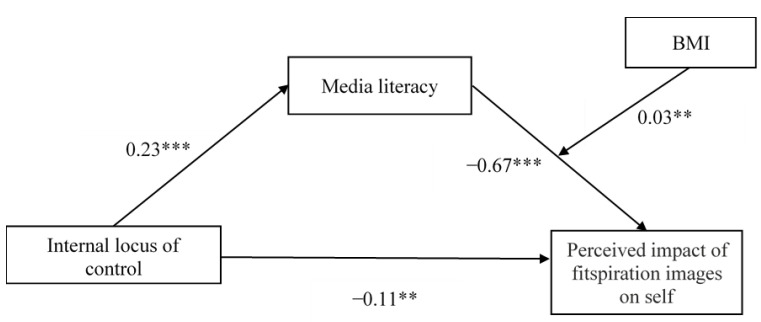
The moderated mediation model of media literacy and BMI while controlling for age, gender and body satisfaction. Note. BMI = Body mass index. ** *p* < 0.01. *** *p* < 0.001.

Figure 2 shows the moderated mediation model. Hypothesis 4 was supported as a significant moderated mediation effect was found, where B = 0.01, SE = 0.003, 95% CI (0.001, 0.01). This result indicated that BMI significantly moderated the indirect effect of locus of control on the perceived impact of fitspiration images on self by buffering the mediating effect of media literacy on the perceived impact of fitspiration images on self. The indirect effect of internal locus of control on the perceived impact of fitspiration images on self via media literacy was significant for participants with low BMI, where B = −0.05, SE = 0.02, 95% CI (−0.09, −0.02), and moderate BMI, where B = −0.03, SE = 0.01, 95% CI (−0.05, −0.01). However, the indirect effect of internal locus of control on the perceived impact of fitspiration images on self via media literacy was found not significant for participants with high BMI, where B = −0.01, SE = 0.01, 95% CI (−0.03, 0.02).

## 5. Discussion

The objectives of this study were to test (1) the direct relationship between internal locus of control and perceived effect of fitspiration images on self; (2) the mediation effect of media literacy between internal locus of control and perceived effect of fitspiration images on self; (3) the moderation effect of BMI on the relationship between media literacy and perceived effect of fitspiration images on self and (4) the moderation effect of BMI on the indirect relationship between internal locus of control and perceived effect of fitspiration images on self through media literacy.

As postulated in H1, internal locus of control was found to be negatively associated with the perceived impact of fitspiration photos on self, suggesting that participants believed that their resistance to fitspiration image effects stemmed from their own internalities. This study concurs with past research which showed that media viewers high on internal locus of control reported lower levels of vulnerabilities to media content [18,19] and lesser nomophobia tendencies [20]. Similarly, this study provides support for Fout and Vaughan’s [84] findings which suggested that having an internal locus of control may help viewers resist television as a source of ideal body modeling. The result also corroborates the premise of attribution theory where the outcomes of an action are attributed to the causal beliefs of internality if the management of the outcomes reflects well on them [16]. The respondents in the present study demonstrated internal attribution because they perceived that handling the outcomes of the events reflects well on their self-image.

The study also confirmed that media literacy mediates the relationship between internal locus of control and the perceived impact of fitspiration images on self (H2). This finding reflects that having the ability to control the events in life is translated into the reporting of media literacy, which in turn helps the viewers of fitspiration images perceive themselves as being safeguarded from the negative effects of such images. This result supported the contention that media literacy empowers individuals by encouraging them to filter and reject unrealistic content [22,25], thus minimizing internalization and upward comparison [24,25,80]. The present study also substantiates that those who assign the causes of the events in life to their own abilities and motives [16,17] perceive themselves as confident and efficient in accomplishing tasks, resisting peer pressure online and evaluating information critically [47,54,85,86].

In addition, the results of the present study showed that the strength of the positive relationship between media literacy and the perceived impact of fitspiration images on self is subject to viewers’ BMI as postulated in H3. In other words, media literacy has a stronger negative influence on the perceived effect of fitspiration images on self among those with low BMI. Indeed, BMI overpowers the influence of media literacy on the percepton of ideal image effects on self. These findings suggest that those with higher BMI are more vulnerable to appearance ideals even though they are media literate. Besides, the results of the present study demonstrated that the moderated mediation model exerts an effect on fitspiration images with lower BMI (H4), signifying the moderating role of high BMI in reducing the negative effect of internal locus of control on the perceived effects of fitspiration images on self through media literacy. By testing BMI as a moderator in the investigation of the perceived influence of ideal images on self, this study contributes to the existing literature on BMI that lower levels of BMI act as a defensive mechanism to reduce the impact of media on the perceived influence of fitspiration images on self. These results provide support for past research which demonstrated the positive influence of high BMI on vulnerability to the negative effects of the ideal bodies displayed in media [66]. Those with high BMI tend to internalize the media body ideals and report higher levels of body dissatisfaction [14,27].

### 5.1. Theoretical Implications

The present research endeavor yielded several theoretical implications. Firstly, this is the first empirical study, to our best knowledge, associating internal locus of control with the perception of media ideals effect in the context of social media, specifically fitspiration photos on Instagram. Secondly, this paper explained the mechanism underlying the association between internal locus of control and the perceived impact of ideal images on self by introducing media literacy as a mediator. Therefore, the mediated model offered a deeper insight into how causal beliefs of internality help individuals navigate themselves to become less vulnerable to ideal appearances on social media. In other words, theoretically, exploring the relationship between internal locus of control and perceived effects of images on self should not be confined to a direct relationship; instead, an indirect and complex relationship would be more fruitful to explain the perceived media effect. Thirdly, a moderated mediation model was developed by including BMI as a moderator which makes the mediation effect of media literacy conditional. The moderated mediation model explains the complexity of the perceived effect of ideal media images by incorporating the three pivotal variables, namely respondents’ predisposition (internal locus of control), critical thinking (media literacy) and physical state (BMI). Finally, this study corroborated the robustness of attribution theory in elucidating the causes attributed to resistance to the effect of ideal bodies on self.

### 5.2. Practical Implications

As for the practical implications, this paper underscores the importance of internal locus of control as a main driver of the perceived effect of media on self even without the mediation of media literacy. This result suggests that SNSs users’ dispositional beliefs, i.e., internal locus of control, can be a booster to resist the content of fitspiration images. Health care professionals and institutions can draw some inspiration from the present paper to devise psycho-educational interventions for SNSs users, especially those who are more vulnerable to fitspiration images, to boost their confidence in their capability of controlling their own life. Internal locus of control should be included as a crucial psychological attribute in health campaigns so that receivers can resist the negative impact of unrealistic body ideal internalization from the prevalent fitspiration images flooding SNSs. As demonstrated in the results, internal locus of control is a fundamental determinant to buffer the perceived impact of fitspiration images on social media directly. It could also advocate for educational organizations to pay more attention to curriculum designs that promote a general internal locus of control. As a result, recipients will be reinforced in their capability, autonomy, and responsibility towards the consequences of viewing the fitspiration images with the socially agreed and defined beauty standards, which are both unrealistic and narrow. A study reported that Malaysian university students hold a moderate level of internal locus of control, suggesting that they have low confidence in their capability to control what they encounter in their life and that they perceive their autonomy, as well as responsibility, are limited by external forces [87]. The moderate level of internal locus of control of Malaysian students should receive sufficient attention from health campaigners because it is not adequate for these young people to defend against and buffer the unrealistic beauty standards embodied in the fitspiration images on SNSs.

Furthermore, the present study illuminates media literacy as a mechanism that accounts for the association between internal locus of control and the perceived impact of fitspiration images on self. This result offers implications for media literacy advocates who believe in the power and influence of intervention programs to enhance social media users’ critical thinking. This study showed that media literacy reduces the perceived impact of fitspiration images on self. In fact, media literacy protects viewers from the allegedly detrimental impacts of fitspiration images by encouraging active and critical participation in evaluating such toxic media culture. Henceforth, media literacy interventions are suggested as the solutions to the negative impact of ideal bodies on SNSs. Not only can it safeguard SNSs users against the ideal bodies internalization by encouraging them to be more critical in their assessment of media content but it can also decrease acceptance of the thin standard of beauty. Factors that determine SNSs users’ self-worth other than body shape and appearance, healthier norms and values related to body and appearance should be highlighted and promoted using hashtags and SNSs users should be encouraged to internalize the healthier values. Media literacy can empower SNSs users to stimulate less desirability to mirror the subjects in fitspiration images and obtain the bodies fitspiration images owners expose. Media literacy is vital in an increasingly dynamic mediated and digitalized society. There is a lack of new media literacy in Malaysia, particularly among young generations [32,33]. This calls for immediate action to create a media literacy education program to improve Malaysian’s new media literacy tailored to empower them to become more critical media consumers and to protect them against fitspiration images prevalent on SNSs, especially the photo-based platform, Instagram.

The moderation results indicate that high BMI can repress the effect of media literacy on the perceived effect of fitspiration images on self. Therefore, to curb the perceived influence of fitspiration images on SNSs, health intervention programs should make known to people of different genders and age groups the scientific healthy BMI standard, and at the same time, reinforce health-related motivation rather than appearance-related motivation for those with higher BMI to lose weight to fit into the healthy BMI range. The importance of fitness and health rather than the socially agreed ideal beauty standard should be the core of these educational programs to promote a healthy lifestyle. As depicted semantically by its name, fitspiration images were originally intended to spread the idea of fitness in their content rather than to promote the ideal appearance to mislead audiences. Therefore, health intervention programs can help SNSs users, especially those with high BMI, to realize that the true intentions of those fitspiration images that they encountered on social media are promoting health and fitness. Consequently, they should also lose weight for their fitness and health. Compared with other Asian countries, Malaysia has one of the highest rates of obesity in the population [29]. Therefore, even though the SNSs users in Malaysia are equipped with media literacy and internal health locus of control, they are still subject to those fitspiration images because of the high prevalence of overweight and obesity in the whole population. That contributes to the need for health policies promoting a healthy lifestyle and practices to achieve a lower BMI value to prevent harm after viewing fitspiration images on Instagram and other SNSs.

### 5.3. Limitations

Although this study has made several contributions, its limitations should also be reflected. This study relied on a cross-sectional survey, and thus it failed to produce inferences of causality [88]. Additionally, Chinese females with an average age of 20 who were conveniently recruited constituted the majority of the sample, thus limiting the generalizability of the results [75]. Therefore, the findings should be validated through further research in other contexts by recruiting a more heterogeneous sample that represents the population. Besides, this study was limited to the self-report of BMI, which might have resulted in the underreporting or overreporting of height and underreporting of weight. Past studies showed that people tend to underestimate their weight and overestimate their height [89,90]. In addition, BMI is getting outdated as a biomarker index, and a body shape index (ABSI) is replacing it across a spectrum of medical fields [91,92,93]. Therefore, future studies would be suggested to use ABSI as a biological measure of a biological state. In addition, there might be some concern about the possible social desirability bias which might refrain participants from being absolutely truthful [75] in reporting their media literacy, internal locus of control and perceived effect of fitspiration images. Future research should consider controlling participants’ social desirability. Past studies showed that body image and body objectification also affect perceived pressure from media [14,27]. Therefore, these constructs may also serve as a moderator to extenuate the effect of media literacy and internal locus of control other than BMI, which would be a fruitful area for further work.

## 6. Conclusions

The present study augments the literature on the perceived effects of appearance ideals on social media by scrutinizing the mediating role of media literacy in the relationship between internal locus of control and the perceived effect of fitspiration images on self. Overall, the results suggest that the internal locus of control influences the perception of fitspiration images’ effects on the self through media literacy. Besides, BMI mitigates the association between media literacy and the perceived effect of fitspiration images on self as well as the mediation effect of media literacy. These results imply that ascertaining the mediating role of media literacy and the moderating role of BMI is beneficial for understanding the process involved in the perceived impact of ideal images on self. This study enhances our understanding of the effect of ideal appearances promoted on social media by extending the existing literature. In addition, the findings bear out the robustness of attribution theory in the context of the influence of ideal images on SNSs.

## Figures and Tables

**Table 1:** Demographic profile of participants (*N* = 321).

**Background information**	***n* (%)**
Gender	
Male	87 (27.1%)
Female	234 (72.9%)
Ethnicity	
Malay	54 (16.8%)
Chinese	195 (60.7%)
Indian	53 (16.5%)
Others	19 (5.9%)
**Background information**	**Mean (*SD*)**
Age	20.93 (2.18)
Height	163.60 (8.09)
Weight	57.55 (12.60)
BMI	21.40 (3.85)

**Table 2:** Means, standard deviation and correlations among variables.

No.	*M (SD*)	1	2	3	4	5	6
1. Age	20.93 (2.18)	1					
2. Body satisfaction	12.20 (3.50)	0.06	1				
3. BMI	21.40 (3.85)	0.13 *	−0.18 **	1			
4. ILOC	29.37 (4.81)	0.05	−0.02	−0.05	1		
5. Media literacy	29.08 (5.31)	0.07	0.08	−0.04	0.21 ***	1	
6. PIFS	12.49 (3.25)	0.03	0.02	−0.08	−0.20 ***	−0.21 ***	1

Note. *N* = 321. BMI = Body mass index. ILOC = Internal locus of control. PIFS = Perceived impact of fitspiration images on self. * *p* < 0.05. ** *p* < 0.01. *** *p* < 0.001.

**Table 3:** The moderated mediating effect of internal locus of control on the perceived effect of fitspiration images on self.

Predictors	On ML			On PIFS		
	*B*	*t*	*p*	*B*	*t*	*p*
Age	0.14	1.05	0.161	0.11	1.37	0.172
Gender	−0.69	−1.06	0.289	−0.52	−1.31	0.193
ILOC	0.23	3.81	<0.001	−0.11	−3.08	0.002
ML				−0.67	−3.65	<0.001
ML x BMI				0.03	3.07	0.002

Note. BMI = Body mass index. ILOC = Internal locus of control. ML = Media literacy. PIFS = Perceived impact of fitspiration images on self.

**Table 4:** Conditional effects of internal locus of control on the perceived effect of fitspiration images on self by BMI.

					95% *CI*	
BMI	Effect	*SE*	*t*	*p*	Lower	Upper
Low	−0.22	0.05	−4.61	<0.001	−0.31	−0.13
Moderate	−0.12	0.03	−3.58	<0.001	−0.19	−0.05
High	−0.02	0.05	−0.49	0.628	−0.11	0.07

Note. BMI = Body mass index.

## Data Availability

The raw data of the present study are available at https://osf.io/arn9m/?view_only=a597e669abeb43a4bc5f203e47ba4ea0, accessed on 28 January 2022.

## Abbreviations

BMI	Body Mass Index
CI	Confidence Interval
MAQ	Media Attitudes Questionnaire
PTSD	Post-traumatic Stress Disorder
SNS	Social Networking Site
SPSS	Statistical Package for the Social Sciences
WHO	World Health Organization
